# Multimodal optical imaging for the assessment of the teratogenic effects of ethanol on zebrafish development

**DOI:** 10.1117/1.JBO.31.6.066004

**Published:** 2026-06-26

**Authors:** Leah A. Lewis, Md Mobarak Karim, Christian Zevallos-Delgado, Mahshid Babeli Nia, Sarah M. Empie, Halpandeniya Hewage Helani N. Alwis, Manmohan Singh, Salavat Aglyamov, Arne C. Lekven, David Mayerich, Kirill V. Larin

**Affiliations:** a University of Houston, Department of Biomedical Engineering, Houston, Texas, United States; b University of Houston, Department of Electrical and Computer Engineering, Houston, Texas, United States; c University of Houston, Department of Biology and Biochemistry, Houston, Texas, United States

**Keywords:** prenatal alcohol exposure, embryonic development, zebrafish, optical imaging, optical coherence tomography, fluorescence microscopy

## Abstract

**Significance:**

Prenatal alcohol exposure is a major cause of neurodevelopmental and growth impairments collectively termed fetal alcohol spectrum disorders (FASD). However, the mechanisms by which ethanol disrupts early embryonic development remain incompletely understood; for example, the potential link between ethanol treatment and biomechanical properties of tissues that lead to defects in development is not understood.

**Aim:**

To address this gap in knowledge, we used zebrafish (*Danio rerio*), a powerful vertebrate model with optical transparency and rapid development, to investigate ethanol-induced alterations during embryogenesis.

**Approach:**

Embryos were exposed to varying concentrations of ethanol and assessed using a multimodal imaging approach integrating light sheet fluorescence microscopy, optical coherence tomography, and optical coherence elastography.

**Results:**

This platform enabled high-resolution, noninvasive, and parallel visualization of structural, molecular, and biomechanical changes. We found that ethanol exposure disrupts Wnt ligand expression in the nervous system, which correlates with a cascade of morphological abnormalities consistently detected across imaging modalities and consistent with alterations to Wnt/β-catenin signaling activity.

**Conclusions:**

These findings highlight the critical role of Wnt/β-catenin signaling in alcohol-induced teratogenesis and underscore the value of zebrafish models and multimodal imaging for advancing our understanding of FASD pathogenesis.

## 1 Introduction

Prenatal alcohol exposure (PAE), defined as exposure to alcohol prior to birth, is a leading cause of neurodevelopmental disorders and physiological birth defects in the United States.^1^^,^^2^ These developmental defects are grouped under the term fetal alcohol spectrum disorders (FASDs) and range from mild neurodivergent outcomes, such as attention deficit hyperactive disorder,^3^^,^^4^ to significant physical impairments such as spina bifida and alterations to brain structure.^4^ FASD is a major public health problem, as the Centers for Disease Control and Prevention (CDC) has estimated that up to 5.0% of children in the U.S. suffer from FASD.^5^ The CDC also reported that the number of women who drink alcohol while pregnant has increased from 9.2% to 14% over the past 14 years.^2^ Given the challenges in diagnosing FASD, this increasing trend in alcohol consumption may lead to underdiagnosis. Although it is understood that PAE results in fetal growth deficits through disrupted cell signaling pathways,^6^ apoptosis of neural cells,^7^ and vasoconstriction,^8^^–^^10^ the exact mechanisms through which alcohol causes these effects are not fully understood. One hypothesis is that PAE disrupts cell division and impairs cell proliferation through the downregulation and upregulation of biological pathways. For example, a study that investigated changes in Wnt signaling following PAE during neurogenesis observed a decrease in Wnt signaling pathways and alterations to Wnt genes.^6^ As Wnt signaling and expression of Wnt genes are crucial to promoting cell proliferation and differentiation during development, the downregulation of Wnt signaling has been hypothesized to disrupt neuronal differentiation, resulting in alcohol-related neurological disorders, which are associated with FASD. To further provide insight into the mechanisms through which alcohol interrupts molecular, structural, and biomechanical pathways during development and gives rise to FASD, we employed zebrafish (*Danio rerio*) as a model for investigating PAE and FASD.

Zebrafish are a well-established model for studying development and toxicology, sharing around 70% of their genes with humans and exhibiting comparable anatomical features.^11^^–^^13^ Zebrafish are highly advantageous vertebrate models due to their ease of handling, genetic manipulability, high fertility, and rapid development, allowing observation of multiple developmental stages within days.^14^ In addition, their optically transparent embryos, which develop *ex-utero*, make them particularly well suited for high-resolution imaging studies. Furthermore, the development of thousands of transgenic lines, in which fluorescent proteins are expressed in molecules, organelles, cells, or tissues, has transformed zebrafish into a powerful and versatile model, accelerating discoveries across diverse fields of biological research.^15^ Over 8000 transgenic lines have been developed, which incorporate green fluorescence protein (GFP) alone.^15^ These transgenic lines have enabled extensive biomedical research in countless areas, including signal transduction, the craniofacial skeletal system, the nervous system, the digestive system, and intracellular organelles.^15^ For example, the most extensively used GFP lines for vasculature research include Tg(kdrl:eGFP),^16^ also referred to as Tg(flk1:eGFP), and Tg(Tie2:eGFP),^17^ in which an enhanced GFP (eGFP) is expressed in vascular endothelial cells, allowing visualization of angiogenesis in zebrafish embryos.

Although various lines of transgenic zebrafish have been utilized in conjunction with fluorescence microscopy to study PAE, very few studies have utilized fluorescent zebrafish embryos to investigate the influence of PAE on Wnt signaling and the expression of Wnt genes. One study utilized several zebrafish lines, Tg(nrd:eGFP), Tg(TP1:GFP), Tg(TP1:mCherry), Tg(gfap:GFP), and Tg(Tcf/Lef-miniP:dGFP), to investigate the effects of ethanol exposure on Wnt/β-catenin signaling via brightfield and confocal fluorescence microscopy imaging of zebrafish eyes.^18^ Although this study showed that ethanol exposure disrupted Wnt signaling, only the impact on retinal cell differentiation was investigated. Additional GFP-derived lines have been developed to enable visualization of Wnt signaling in zebrafish embryos, such as Tg(top:GFP) and Tg(7xTCF-Xla. Siam:GFP),^15^^,^^19^ however, their implementation has not been extended to PAE research. As Wnt/β-catenin signaling is crucial for brain formation and posteriorization,^20^ it is important to understand the impact that ethanol has on Wnt/β-catenin signaling in relation to morphogenesis. Here, we present another line that enables phenotypic evaluation of Wnt ligand expression during embryonic development through the expression of GFP throughout the entire brain and spinal cord of developing zebrafish embryos. In $\mathrm{Tg}(zf\mathrm{CNE}20;\mathrm{EGFP}:\mathrm{wnt}1-5.1\mathrm{wnt}10b:\mathrm{mRFP};zf\mathrm{CNE}20{)}^{g7}$, hereafter $\mathrm{Tg}(\mathrm{ALW}11{)}^{g7}$, zebrafish embryos, expression of GFP is a marker for *wnt1* and *wnt10b*, expressing cell lineages. Here, zfCNE20 is a conserved noncoding element, which interacts with promoters to regulate the expression of *wnt1* and *wnt10b*^21^ and thereby GFP. Utilizing this transgenic line enabled visualization of molecular changes resulting from PAE, through the expression of GFP in the anteroposterior central nervous system (CNS) of the zebrafish embryos, which is divided into the forebrain, midbrain, hindbrain, and spinal cord.^20^ Furthermore, as expression of *wnt1* and *wnt10b* is critical for activation of Wnt/β-catenin signaling pathway,^22^ utilizing $\mathrm{Tg}(\mathrm{ALW}11{)}^{g7}$ zebrafish allowed us to observe the impact of ethanol on the Wnt/β-catenin signaling pathway, which was phenotypically characterized by the absence or presence of GFP expression.

Zebrafish are a widely used model for assessing the effects of acute ethanol exposure on embryonic development and understanding the mechanisms of FASD.^23^ A large majority of studies that use zebrafish as a model for FASD utilize traditional light microscopy methods, such as stereomicroscopes and confocal microscopes, as a primary tool for imaging and analysis of defects following ethanol exposure.^24^^–^^27^ Although microscopy has become an invaluable tool in biological studies, traditional microscopy methods are incapable of depth-resolved imaging or optical sectioning. Therefore, capturing information of entire organisms requires continuously rotating the sample to acquire images from various planes, which can become time-consuming when investigating large sample groups. An optical imaging method that overcomes these limitations is optical coherence tomography (OCT). OCT is a noninvasive 3D imaging technique that enables visualization of biological specimen microstructure with a typical resolution of ∼10  μm.^28^^,^^29^ OCT uses low-coherence interferometry to measure the time delay and intensity of light backscattered within a sample, creating an image representative of the distribution of microstructures throughout the sample.^28^ A series of single scans in depth (A-scans) is acquired as the OCT beam sweeps across the sample, which is combined into a 2D cross-sectional image (B-scan), which is representative of one slice throughout the sample.^29^ Acquiring a series of B-scans enables 3D reconstruction of the whole sample. Furthermore, OCT is highly suitable for imaging zebrafish embryos. Although zebrafish embryos appear transparent under traditional light microscopy, they possess sufficient internal scattering to generate high-contrast OCT images. OCT was originally developed for ophthalmology,^30^^,^^31^ but its features, such as its spatial resolution, imaging speed, label-free imaging, and functional extensions, have propelled OCT as a prominent technique in developmental biology.^32^^–^^35^ Over the past decade, OCT has demonstrated its power for researching embryonic development in zebrafish.^36^ Although OCT imaging of zebrafish has been widely implemented in neurological, developmental, and retinal studies, there is a lack of imaging studies for investigating ethanol-induced defects in zebrafish embryos. Furthermore, existing research only evaluates ethanol-induced effects after 72 h post-fertilization (hpf).^37^ In zebrafish embryos, the segmentation period (10 to 24 hpf) is characterized by rapid neural growth and differentiation into the forebrain, midbrain, and hindbrain.^38^ Exposure to ethanol during this period has a major impact on the development of the brain, eyes, and other organs,^39^ and morphological changes observed at later stages often stem from developmental disruptions during this crucial period. Using OCT to assess zebrafish embryos immediately after this critical developmental stage provides an opportunity to increase our understanding of the genesis of ethanol-induced developmental defects.

Although OCT and traditional microscopy methods have been widely utilized for assessing the structural development of zebrafish embryos, they are unable to provide direct molecular information in 3D.^40^ To overcome this limitation, many researchers have utilized confocal fluorescence microscopy to image the brain,^41^ vasculature,^42^^,^^43^ and whole embryo development in transgenic zebrafish models.^24^ Although the introduction of confocal fluorescence microscopy has transformed biomedical research, this technique is not without limitations. Smaller pinholes correspond to better rejection of out-of-focus light. However, as the pinhole size decreases, an increase in laser illumination power is required to achieve the same image quality.^44^ Although the pinhole restricts the amount of light reaching the detector, it does not restrict the light that reaches the sample, which can lead to photobleaching and phototoxicity.^45^ A fluorescence technique that overcomes these limitations is light sheet fluorescence microscopy (LSFM). Instead of using a pinhole, LSFM utilizes a thin sheet of light to sequentially illuminate planes throughout the sample, enabling improved optical sectioning and volumetric imaging.^46^ The use of selective illumination reduces the risk of photobleaching by only exciting the fluorescence within each plane that is imaged. Like confocal microscopy, LSFM has been implemented in zebrafish studies, which focus on the neural,^47^ cardiac,^48^ vasculature,^47^ and whole embryo development.^49^^,^^50^ Furthermore, the reduced risk of phototoxicity and photobleaching makes LSFM an attractive tool for long-term imaging of embryonic development.^49^

Although LSFM has been extensively applied in zebrafish developmental research, there remains a significant gap in its application for investigating ethanol-induced defects in embryos. Current studies assessing ethanol-induced developmental abnormalities primarily rely on fluorescence stereomicroscopy,^51^ epifluorescence microscopes,^52^ or confocal fluorescence microscopy,^18^^,^^24^^,^^51^^,^^53^ which are limited to two-dimensional imaging, not capable of optical sectioning, or pose a greater risk of photodamage to the sample. By contrast, LSFM offers rapid, high-resolution, three-dimensional visualization of live embryos with minimal phototoxicity, making it uniquely suited for capturing subtle structural and morphological changes that may be overlooked with conventional techniques. Applying LSFM to the study of ethanol-induced developmental defects in zebrafish embryos has the potential to provide comprehensive, volumetric insights into the spatial distribution, severity, and progression of ethanol-related abnormalities. Using $\mathrm{Tg}(\mathrm{ALW}11{)}^{g7}$ in conjunction with LSFM will enable visualization of the impact of ethanol on Wnt/β-catenin signaling, which directly influences the development of the posterior neural plate.^20^

The combination of OCT and LSFM has the potential to fill a gap in understanding the effects of ethanol on structural and molecular disruptions during embryonic development, but mechanical processes also play a crucial role in embryonic development.^54^^–^^57^ Examples of these processes include gastrulation,^58^ neurulation and lumen formation,^56^^,^^57^ the morphogenesis of organs,^59^ and convergent extension.^60^ Understanding these mechanical processes is essential not only for elucidating normal embryonic development but also for studying the origins of congenital abnormalities. For example, neural tube defects such as spina bifida and heart defects often stem from mechanical disruptions during neurulation and lumen formation.^59^^,^^61^ Furthermore, mechanical processes related to cell differentiation, gene expression, and shaping of tissues and organs are highly integrated with biological signals,^62^ demonstrating the need to integrate mechanical assessment with structural and biomolecular assessment to uncover how mechanical disruptions stemming from PAE contribute to physical and cognitive abnormalities.^63^ Clinically used methods for mechanical assessment of biological tissues include magnetic resonance elastography (MRE) and ultrasound elastography (USE). Although these methods have been used in mapping the mechanical properties of adult zebrafish,^64^ they lack the resolution needed for zebrafish embryos.^65^ Other methods of mechanical characterization and imaging, which have finer resolutions, have been employed in assessing the biomechanical properties of zebrafish embryos and larvae, such as Brillouin microscopy,^66^^–^^68^ optical tweezers,^69^^,^^70^ and atomic force microscopy (AFM).^71^^,^^72^ However, their long imaging times limit the quantity of embryos that can be mapped in a timely manner, as well as the ability to map entire embryos. One method that overcomes these limitations is optical coherence elastography (OCE). OCE is the functional extension of OCT, which commonly utilizes compression-based or wave-based methods to map tissue mechanical properties.^73^^–^^76^ The resolution and speed of OCE are optimal for mapping the biomechanical properties of whole embryos in 3D in a matter of minutes,^77^ or even seconds, with ultrafast methods.^78^^,^^79^ Traditional wave-based elastography methods are not able to capture wave propagation reliably in highly heterogeneous embryos due to the presence of numerous reflections. However, in reverberant shear wave optical coherence elastography (Rev-OCE), these reflections can be leveraged for high-resolution mechanical imaging,^80^ with a mechanical resolution and contrast that surpass those of traditional wave-based OCE.^81^ The scattering of zebrafish embryos is suitable for mechanical characterization by OCE, which provides unique capabilities for rapid, noninvasive 3D mapping of stiffness of distinct regions, such as the ventricles and midbrain. Overall, the ability to map biomechanical changes is a vital capability for our study, as ethanol-derived developmental defects often manifest as biomechanical alterations before anatomical changes become apparent.^82^^,^^83^ Integrating mechanical assessment by Rev-OCE in addition to structural and molecular imaging allowed us to perform unique quantitative assessments of ethanol-induced developmental changes that often remain undetected or are unmeasurable through imaging alone, providing a more complete picture of ethanol-induced developmental abnormalities.

In this study, we investigated the effects of prenatal alcohol exposure by subjecting zebrafish embryos to varying concentrations of ethanol. We employed a synergistic imaging platform, incorporating LSFM, OCT, and OCE, to provide a comprehensive analysis of zebrafish development across structural, molecular, and mechanical scales. Imaging with OCT and LSFM was performed using our home-built system, which integrates OCT and LSFM (hereafter, OCT-LS), enabling simultaneous capture of structural and molecular changes during embryonic development in the same plane.^84^ Following imaging with OCT-LS, measurements were performed by Rev-OCE on a separate OCT system. LSFM provided high-speed, 3D visualization of specific molecular markers and cell populations. OCT complemented this by offering label-free, high-resolution structural morphological imaging. OCE added a critical functional dimension by quantifying tissue biomechanical properties, i.e., stiffness. We observed ethanol-induced disruptions to the molecular, structural, and biomechanical properties of zebrafish embryos. LSFM images of zebrafish embryos displayed altered expression of GFP throughout the CNS, which was indicative of disrupted *wnt1* and *wnt10b* gene expression, and thereby disrupted Wnt/β-catenin signaling. OCT images and Rev-OCE maps displayed morphological defects and softening of zebrafish embryos, respectively, in which the severity increased in a dose-dependent manner. The results obtained through OCT-LS and Rev-OCE all demonstrated the presence of ethanol-induced defects, which are consistent with disrupted Wnt/β-catenin signaling. Overall, this integrated approach enabled high-resolution, quantitative evaluation of the structural, molecular, and biomechanical alterations that arise from ethanol exposure, providing a powerful platform for parallel, noninvasive analysis of alcohol-induced developmental disruptions and highlighting the utility of zebrafish as biological models for FASD. Furthermore, this multiparametric approach provided a more holistic understanding of the teratogenic effects of ethanol than any single imaging modality could offer alone. To our knowledge, this study represents the first application of an integrated LSFM, OCT, and OCE imaging approach to provide a holistic understanding of the teratogenic effects of ethanol on embryonic development.

## 2 Materials and Methods

### 2.1 Alcohol Exposure

All animal experiments in this study were approved by the University of Houston Institutional Animal Care and Use Committee (IACUC). Embryos were collected from wild type (NHGRI) or $\mathrm{Tg}(\mathrm{ALW}11{)}^{g7}$ outcrosses and incubated in their culture dishes containing fish water at 29°C. At 6 h post-fertilization (hpf), the health of the embryos was checked with a brightfield microscope (Stemi 508, Carl Zeiss AG, Germany). Embryos that exhibited gastrulation arrest or coagulation were separated and disposed.

**Fig. 1:**
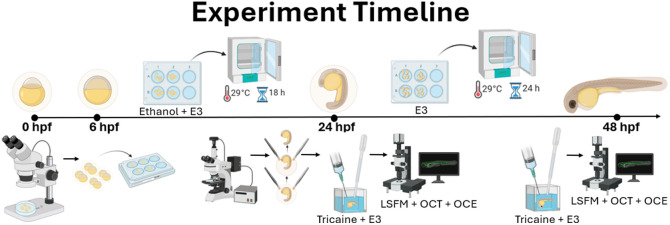
Experimental timeline. Zebrafish embryos were exposed to various concentrations of ethanol dissolved in E3 embryo media (0.0% EtOH as a control, 1.0% EtOH, 1.5% EtOH, and 2.0% EtOH). At 24 and 48 hpf, embryos were dechorionated and imaged with the OCT-LS system, and mechanical properties of the embryos were assessed with OCE. This figure was created with BioRender.

Groups of 20 to 40 embryos per well were exposed to pure ethanol (EtOH) dissolved in E3 media (5 mM NaCl, 0.17 mM KCl, 0.33 mM ${\mathrm{CaCl}}_{2}$, and 0.33 mM ${\mathrm{MgSO}}_{4}$ in distilled water) in a standard 6-well plate, with each well holding a volume of 3 mL (Fig. 1). Concentrations of 1.0%, 1.5%, and 2.0% EtOH were studied, whereas the control group was exposed to E3 media only. Previous ethanol exposure studies have reported that a dose of 1.0% ethanol roughly corresponds to a blood alcohol concentration of 0.2%, which is equivalent to a binge dose in humans.^85^ In this work, we only modeled binge exposure to ethanol. Embryos were placed in an incubator set to 29°C and incubated in their respective wells from 6 hpf until 24 hpf. At 24 hpf, zebrafish embryos were transferred to new culture dishes containing fresh E3 media. The embryos’ health was checked again utilizing the brightfield microscope, and mortality data were recorded for each experimental group (see the Discussion section in the Supplementary Material). Deceased embryos were separated and properly discarded.

### 2.2 Imaging

**Fig. 2:**
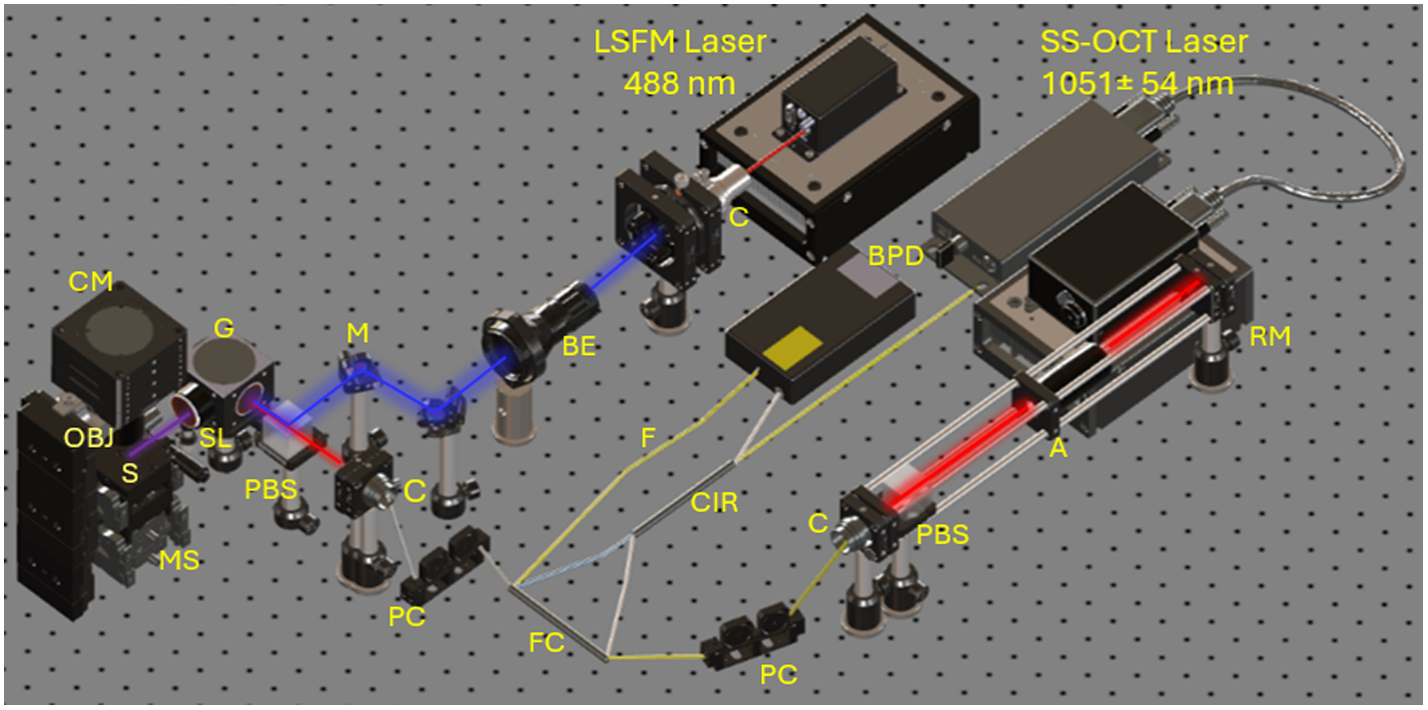
Schematic of the OCT-LS system. A, aperture; BPD, balanced photo detector, BE, beam expander; C, collimator; CIR, circulator; CM, camera; F, fibers; FC, fiber coupler; G, galvanometer; M, mirror; MS, motorized stage; OBJ, objective; PBS, polarization beam splitter; PC, polarization controller; RM, reference mirror; S, sample position; SL, scan lens. The LSFM beam path is shown in blue. The OCT beam path is shown in red. The combined beam path is shown in purple. This figure was created with 3DOptix.

Healthy embryos were imaged with an epifluorescence microscope (Axioscope 5, Carl Zeiss AG) to identify and isolate fluorescent embryos. As fluorescence expression was a requirement for imaging with LSFM, only fluorescent embryos were utilized for imaging with the OCT-LS system (Fig. 2). A 20× stock solution of tricaine (MS-222, TMS, tricaine methanesulfonate) was obtained to anesthetize the fish during imaging and diluted to 1× before use. Fluorescent zebrafish embryos were anesthetized to prevent movement during imaging by being transferred to culture dishes containing 0.2% tricaine in E3 media.^49^ The chorion was manually removed from the embryos to enable clear visualization while imaging. Samples were then inserted into a glass cuvette, which was mounted on the sample stage for imaging.

The zebrafish embryos were first imaged with the OCT-LS system (Fig. 2).^86^ The LSFM sub-system is comprised of a continuous wave 488 nm laser (iChrome MLE, Toptica Photonics Inc., Farmington, New York, United States) for exciting GFP. The fluorescent light emitted from the sample was collected by a water-dipping 16× immersion objective (N16LWD-PF, Nikon Corp., Tokyo, Japan), with a numerical aperture of 0.8 and working distance of 3 mm. Once collected, the emitted fluorescent light passed through a bandpass filter with a central wavelength of 520 nm and a bandwidth of 10 nm, followed by an infinity corrected tube lens to be imaged onto a camera (C11440-22CU, Hamamatsu, Hamamatsu City, Japan). The LSFM-subsystem had a transverse resolution of 2.1  μm, a light-sheet beam thickness of ∼11  μm, and a field of view of 1.81  mm×1.81  mm.^86^

The OCT sub-system was based on a Michelson-style interferometer and was comprised of a swept source laser (Model 1051 SSOCT, Axsun Tech., Billerica, Massachusetts, United States) with a central wavelength of 1051 nm and bandwidth of 109 nm. The sweep rate of the laser was 100 kHz, and the incident power on the sample was ∼6  mW. The OCT sub-system had a sensitivity of 100.1 dB and a sensitivity roll-off of 6.9 dB over 4.1 mm. The system had an axial resolution of ∼7.6  μm and a lateral resolution of ∼14.9  μm. The OCT and LSFM beams were combined utilizing a polarizing beam splitter for concurrent and co-planar imaging.^84^^,^^86^

Rev-OCE imaging was performed on the same embryos after imaging with OCT-LS using a previously published system and procedure.^77^ The OCE system was based on a spectral-domain OCT system and utilized a superluminescent diode (S-840-B-I-20, Superlum Diodes Ltd., Ireland) with a central wavelength of 840 nm and a bandwidth of 49 nm. The OCT system had an axial resolution of 9  μm and a lateral resolution of 8  μm in air. The imaging range of the system in air was 3 mm, and the displacement stability was 0.3 nm at an OCT signal-to-noise ratio >40  dB. The line rate of the camera was 25 kHz during Rev-OCE imaging. The spectral interference signals were captured by a spectrometer (CS800-840/120-250-OC2K-CL, Wasatch Photonics Inc., Morrisville, North Carolina, United States). A piezoelectric bender (BA4510, PiezoDrive, Callaghan, NSW, Australia) was attached to a 5 mm-thick optical window, which served as the sample holder and excited the reverberant shear field. The piezoelectric bender was driven by a 1 kHz sinusoidal signal generated by a function generator (DG4162, Rigol Technologies, Suzhou, China) and amplified by a low-noise power amplifier (PDu150, PiezoDrive, Newcastle, Australia). The zebrafish embryos were placed on the optical window, which was covered with E3 media. The lateral field of view was 2.0  mm×2.0  mm based on the size of the zebrafish embryos. Rev-OCE data processing followed the typical Rev-OCE processing methodology,^81^ where the kernel processing size was 0.4  mm×0.4  mm for the zebrafish. Axial particle velocities were estimated from the temporal phase differences, and the local wavenumber was determined by fitting the 2D autocorrelation function to the analytical solution of the reverberant shear wave model, thereby generating shear wave speed maps.^81^ The average speeds in the brain region, which was chosen based on the OCT structural image, were then used for further quantification and analysis. A total of four zebrafish were imaged for each exposure group at 24 and 48 hpf.

### 2.3 Morphological Assessment

For the morphological assessment of zebrafish embryos’ response to the various concentrations of ethanol, 3D reconstructions of OCT and LSFM data were analyzed in Imaris (Oxford Instruments, Abingdon, United Kingdom). Manual measurements were performed for each sample to measure changes in body length, notochord length, tail curvature, and eye volume (Fig. S1 in Supplemenetary Material). Pericardial edema was also observed among a fraction of the embryos exposed to EtOH. For a detailed procedure for quantifications, see Fig. S1 and Eqs. (S1) and (S2) in the Supplementary Material.

### 2.4 Statistical Analysis

Measurements as a function of ethanol concentration were analyzed for statistical significance using one-way ANOVA with a significance threshold of p-value <0.05. *Post-hoc* pairwise analysis was performed with Bonferroni correction for multiple comparisons, where adjusted p-values <0.05 were considered statistically significant.

## 3 Results

### 3.1 LSFM

**Fig. 3:**
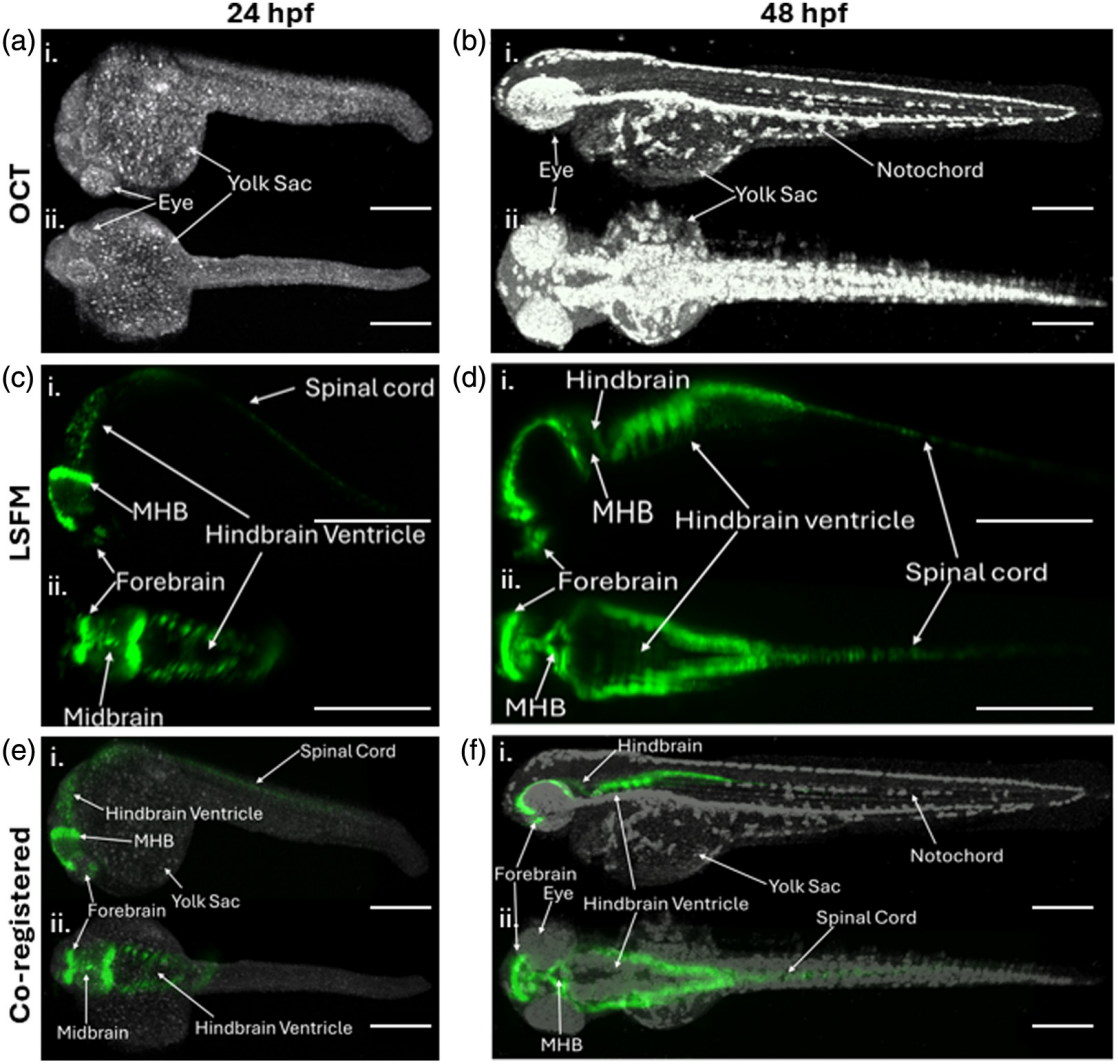
OCT and LSFM images of normal $\mathrm{Tg}(\mathrm{ALW}11{)}^{g7}$ zebrafish embryos. Subfigures show (i) lateral and (ii) dorsal views of embryos imaged with (a)–(b) OCT and (c)–(d) LSFM at 24 and 48 hpf, along with (e)–(f) co-registered images. Images are maximum intensity projections (MIPs) of 3D LSFM and OCT images. All scale bars are 300  μm.

Utilizing LSFM, we examined the changes in Wnt gene expression via the expression of GFP in response to ethanol exposure. In $\mathrm{Tg}(\mathrm{ALW}11{)}^{g7}$ zebrafish embryos, the expression of GFP is indicative of *wnt1* and *wnt10b* gene expression. Furthermore, as *wnt1* and *wnt10b* expression are critical for activation of the Wnt/β-catenin signaling pathway, GFP expression may reflect domains of active Wnt signaling. Expression of *wnt1* and *wnt10b* begins at the 16-somite stage, corresponding to 16 hpf.^87^ Therefore, $\mathrm{Tg}(\mathrm{ALW}11{)}^{g7}$ zebrafish begin to express GFP by 16 hpf. This period is characterized by the formation of the midbrain-hindbrain boundary (MHB) and activation of Wnt/β-catenin signaling. During the pharyngula stage (24 to 48 hpf), Wnt/β-catenin signaling is active throughout the spinal cord,^88^ and expression of GFP extends throughout the entire CNS (Fig. S2 in the Supplementary Material). Prior to exposing zebrafish embryos to ethanol, imaging with OCT-LS was performed on normal $\mathrm{Tg}(\mathrm{ALW}11{)}^{g7}$ zebrafish embryos at 24 and 48 hpf to establish a basis for structural and molecular images (Fig. 3). To validate the fluorescence expression in the zebrafish embryos, LSFM images were compared with *in situ* hybridizations of $\mathrm{Tg}(\mathrm{ALW}11{)}^{g7}$ zebrafish embryos at 24 and 48 hpf (Fig. S2 in the Supplementary Material), which indicated identical regions of *wnt1* and *wnt10b* gene expression.

**Fig. 4:**
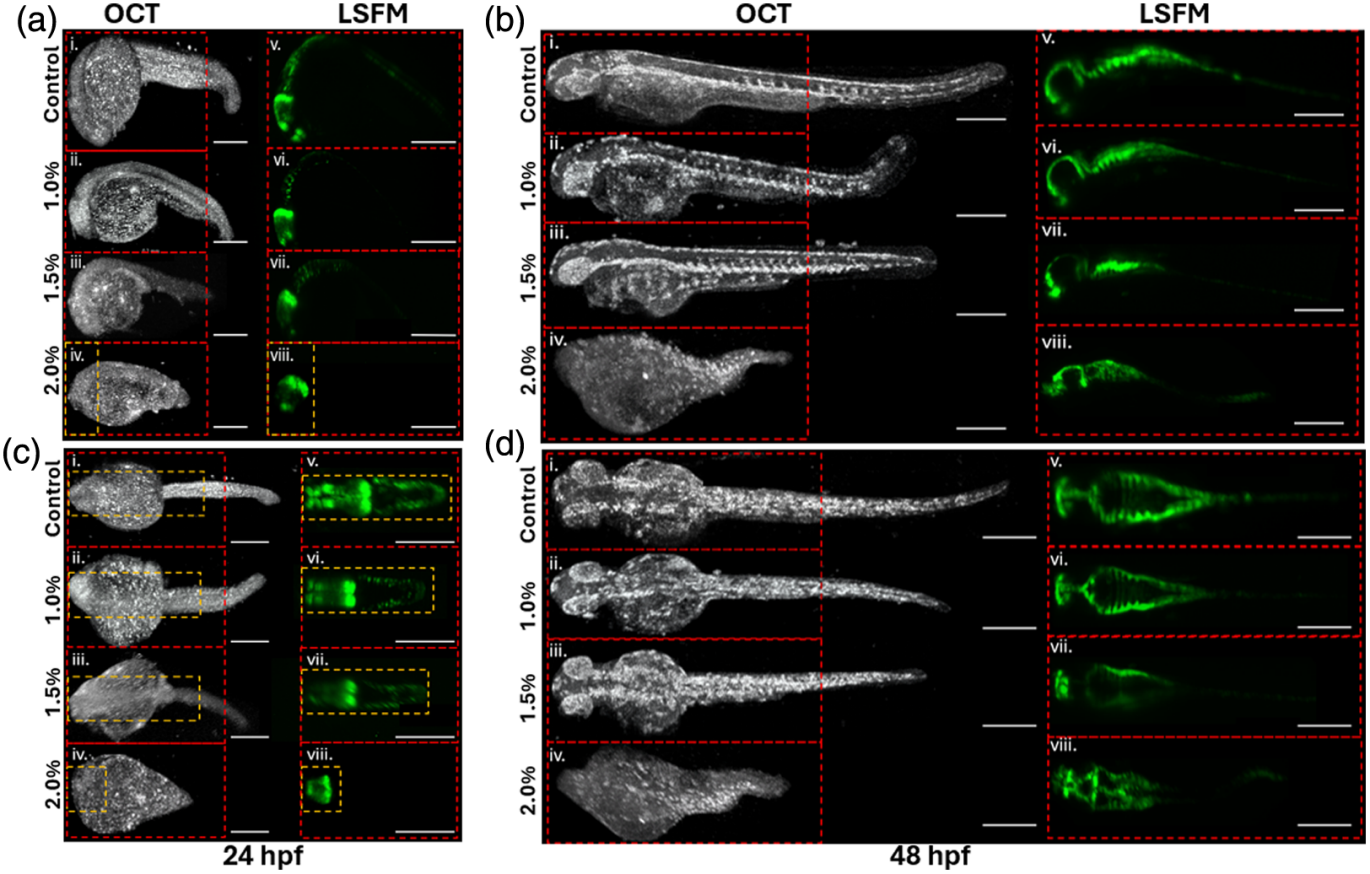
OCT and LSFM images of zebrafish embryos at 24 and 48 hpf. (a)–(b) Lateral and (c)–(d) dorsal views of embryos at (a), (c) 24 hpf and (b), (d) 48 hpf. Subfigures show the (i-iv) OCT and (v-viii) LSFM images for each ethanol exposure concentration. Images are MIPs of 3D LSFM and OCT images. The red dashed rectangles denote the LSFM field of view. The yellow dashed rectangles denote the areas in LSFM images in relation to the OCT images in samples with significant defects. The results displayed in the figure are derived from parallel experiments and show the same embryo at each time point. All scale bars are 300  μm.

As observed through LSFM at 24 hpf [Figs. 3(a), 4(a.v), and 4(c.v)], embryos in the control group demonstrated prominent expression of GFP within the forebrain, midbrain, and hindbrain regions, which extended throughout the spinal cord. After exposure to 1.0% EtOH, embryos still exhibited prominent fluorescence throughout the entire brain; however, there was a decrease in fluorescence expression throughout the spine [Figs. 4(a.vi) and 4(c.vi)]. Exposure to 1.5% EtOH [Fig. 4(a.vii) and 4(c.vii)] resulted in an even greater decrease in the fluorescence observed in comparison to the control group. For embryos exposed to 2.0% EtOH, only fluorescence within the midbrain and hindbrain was observed, whereas no fluorescence was observed in the forebrain or throughout the hindbrain ventricle and spine (Figs. 4(a.viii) and 4(c.viii)].

All zebrafish embryos were re-imaged at 48 hpf for evaluation of development in response to ethanol exposure. Lateral views of the embryos at 48 hpf demonstrated similar fluorescence expression between the control and 1.0% EtOH group, whereas the 1.5% and 2.0% EtOH groups displayed a decrease in fluorescence [Fig. 4(b.v-viii)]. Dorsal views also show decreased fluorescence in embryos exposed to 1.5% EtOH [Fig. 4(d.vii)] and 2.0% EtOH [Fig. 4(d.viii)], along with abnormal development of the hindbrain ventricle.

### 3.2 OCT

Utilizing OCT, we examined the morphological effects of ethanol on the development of the zebrafish embryos, and structural defects were clearly visible (Fig. 4). Quantifications included measurements of body length, notochord length, eye volume, and the degree of tail curvature (Fig. S1 in the Supplementary Material). OCT images of zebrafish embryos at 24 hpf showed a reduction in body size resulting from ethanol exposure in a dose-dependent manner [Figs. 4(a.i-iv) and 4(c.i-iv)]. Zebrafish embryos at 24 hpf still lacked prominent physical features, so all quantifications were performed on the reconstructions of the embryos at 48 hpf only.

**Fig. 5:**
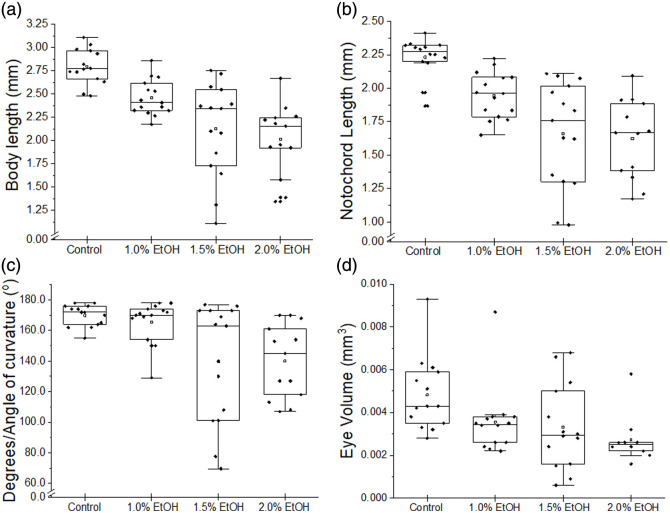
Quantification from OCT reconstructions reveals that ethanol (EtOH) exposure results in significant structural defects in zebrafish embryos. EtOH exposure leads to (a) a reduction in body length, (b) a reduction in notochord length, (c) an increased tail curvature, and (d) a reduction in eye volume at higher concentrations. Control: n=14; 1.0%: EtOH n=16; 1.5%: EtOH n=16; and 2.0%: EtOH n=13. Statistical significance is indicated in Table 1. Data are presented as box plots, where the box is the interquartile range, the whiskers are the 25th and 75th percentiles, the central line is the median, the inscribed box is the mean, and outliers are shown as individual points.

For embryos within the control group (Figs. 4(b.i) and 4(d.i)], the average body length of the zebrafish embryos was 2.79±0.18  mm. Within the 1.0%, 1.5%, and 2.0% EtOH groups, the average body lengths were 2.46±0.18  mm, 2.13±0.48  mm, and 2.02±0.37  mm, respectively. Analysis by one-way ANOVA revealed that there was a significant dependence of body length on the concentration of alcohol (F=14.470, df=3, p<0.001). These results are shown in Fig. 5(a). Post-hoc pairwise analysis with Bonferroni correction revealed that in comparison to the control group, the changes in body length for the 1.0% EtOH group [Figs. 4(b.ii) and 4(d.ii)] were not significant (t=2.628, p=0.067), whereas the changes in body length for the 1.5% EtOH (t=5.220, p<0.001) [Figs. 4(b.iii) and 4(d.iii)] and 2.0% EtOH (t=5.876, p<0.001) [Figs. 4(b.iv) and 4(d.iv)] groups were statistically significant. In comparison to the 1.0% EtOH group, the changes in body length among the 1.5% EtOH group (t=2.637, p=0.066) were not significant, whereas the observed changes in comparison to the 2.0% EtOH group (t=3.394, p=0.008) were significant. Last, when comparing the 1.5% EtOH and 2.0% EtOH groups (t=0.853, p=1), no significant changes were observed.

Due to the significant decrease in body length from ethanol exposure, we also expected a significant decrease in notochord length. The notochord is an elastic, rod-like structure composed of glycoproteins and collagen fibers that serves to protect and support the spinal cord.^89^ At 24 hpf and 48 hpf, the notochord makes up a significant portion of the embryo.^90^ In the control group, the notochord had an average length of 2.23±0.14  mm. For the 1.0%, 1.5%, and 2.0% EtOH groups, the average notochord lengths were 1.95±0.17  mm, 1.66±0.38  mm, and 1.62±0.28  mm, respectively. Analysis by means of one-way ANOVA revealed that there was a statistically significant dependence of notochord length on the concentration of alcohol that the fish were exposed to (F=15.323, df=3, p<0.001). The results are shown in Fig. 5(b). Post-hoc analysis via Bonferroni correction revealed a significant reduction in notochord length in the 1.0% EtOH group compared with controls (t=2.841, p=0.0382), with more pronounced differences in the 1.5% (t=5.636, p<0.001) and 2.0% (t=5.832, p<0.001) EtOH groups. Furthermore, comparison among the other experimental groups revealed that in comparison to the 1.0% EtOH group, the difference in notochord length for both the 1.5% EtOH (t=2.893, p=0.033) and 2.0% EtOH (t=3.142, p=0.016) groups was significant. Last, when comparing the 1.5% EtOH and 2.0% EtOH groups (t=0.354, p=1), no significant difference was observed.

Previous studies have demonstrated that malformation of the notochord can lead to vertebrate spinal defects such as scoliosis.^12^^,^^26^^,^^27^ We observed similar structural abnormalities in the EtOH-exposed groups. In the control group, the average tail curvature angle was 169.9±6.8  deg. For the 1.0%, 1.5%, and 2.0% EtOH groups, the average tail curvature angles were 165.5±13.2  deg, 139.7±37.3  deg, and 140.1±23.3  deg, respectively. Analysis by one-way ANOVA revealed that there was a significant dependence in tail curvature on the concentration of alcohol exposure (F=6.267, df=3, p<0.001). The results are shown in Fig. 5(c). Post-hoc pairwise analysis with Bonferroni correction revealed that in comparison to the control group, the difference in tail curvature for the 1.0% EtOH group was not significant (t=0.486, p=1), whereas the differences in tail curvature compared with the 1.5% EtOH (t=3.344, p=0.009) and 2.0% EtOH (t=3.183, p=0.015) groups were significant. Furthermore, in comparison to the 1.0% EtOH group, the difference in tail curvature for both the 1.5% EtOH t=2.908, p=0.032) and 2.0% EtOH (t=2.759, p=0.048) groups was significant. Last, when comparing the 1.5% EtOH and 2.0% EtOH groups (t=0.0439, p=1), no significant difference was observed.

Previous studies have reported decreases in eye diameter, intraocular distance, and cyclopia resulting from embryonic ethanol exposure.^25^^,^^53^^,^^91^ To determine the effect of ethanol exposure on eye development, we quantified the average eye volume for each zebrafish embryo utilizing the volumetric reconstructions of the OCT data [Fig. S1(a) in the Supplementary Material].^92^ For embryos within the control group, the average eye volume was $0.0048\pm 0.0017\;{\mathrm{mm}}^{3}$. Within the 1.0%, 1.5%, and 2.0% EtOH groups, the average eye volumes were $0.0036\pm 0.0015\;{\mathrm{mm}}^{3}$, $0.0033\pm 0.0019\;{\mathrm{mm}}^{3}$, and $0.0027\pm 0.0011\;{\mathrm{mm}}^{3}$, respectively. Analysis by a one-way ANOVA revealed that there was a significant dependence of eye volume on the concentration of alcohol exposure (F=3.486, df=3, p=0.023). These results are shown in Fig. 5(d). Pairwise post-hoc analysis with Bonferroni correction revealed that in comparison to the control group, the differences in eye volume for the 1.0% EtOH (t=2.007, p=0.303) and 1.5% EtOH (t=2.390, p=0.125) groups were not significant, whereas the difference in eye volume compared to the 2.0% EtOH group (t=3.009, p=0.025) was significant. In comparison to the 1.0% EtOH group, the difference between the 1.5% EtOH group (t=0.383, p=1) and 2.0% EtOH group (t=1.772, p=1) was not significant. Last, when comparing the 1.5% EtOH and 2.0% EtOH groups (t=0.827, p=1), no significant difference was observed.

**Table 1:** Statistical significance of all morphological changes resulting from ethanol exposure.

	Control versus 1.0% EtOH	Control versus 1.5% EtOH	Control versus 2.0% EtOH	1.0% EtOH versus 1.5% EtOH	1.0% EtOH versus 2.0% EtOH	1.5% EtOH versus 2.0% EtOH
**Body length**	p=0.067	p<0.001	p<0.001	p=0.066	p=0.008	p=1
**Notochord length**	p=0.0382	p<0.001	p<0.001	p=0.033	p=0.016	p=1
**Tail curvature**	p=1	p=0.009	p=0.015	p=0.032	p=0.048	p=1
**Eye volume**	p=0.303	p=0.125	p=0.025	p=1	p=1	p=1
**Wave speed** **(24 hpf)**	p=0.559	p=0.283	p=0.001	p=1	p=0.257	p=0.051
**Wave speed** **(48 hpf)**	p=0.122	p=0.001	p<0.0001	p=0.127	p=0.0025	p=0.304

### 3.3 OCE Shows Alteration to Tissue Properties

**Fig. 6:**
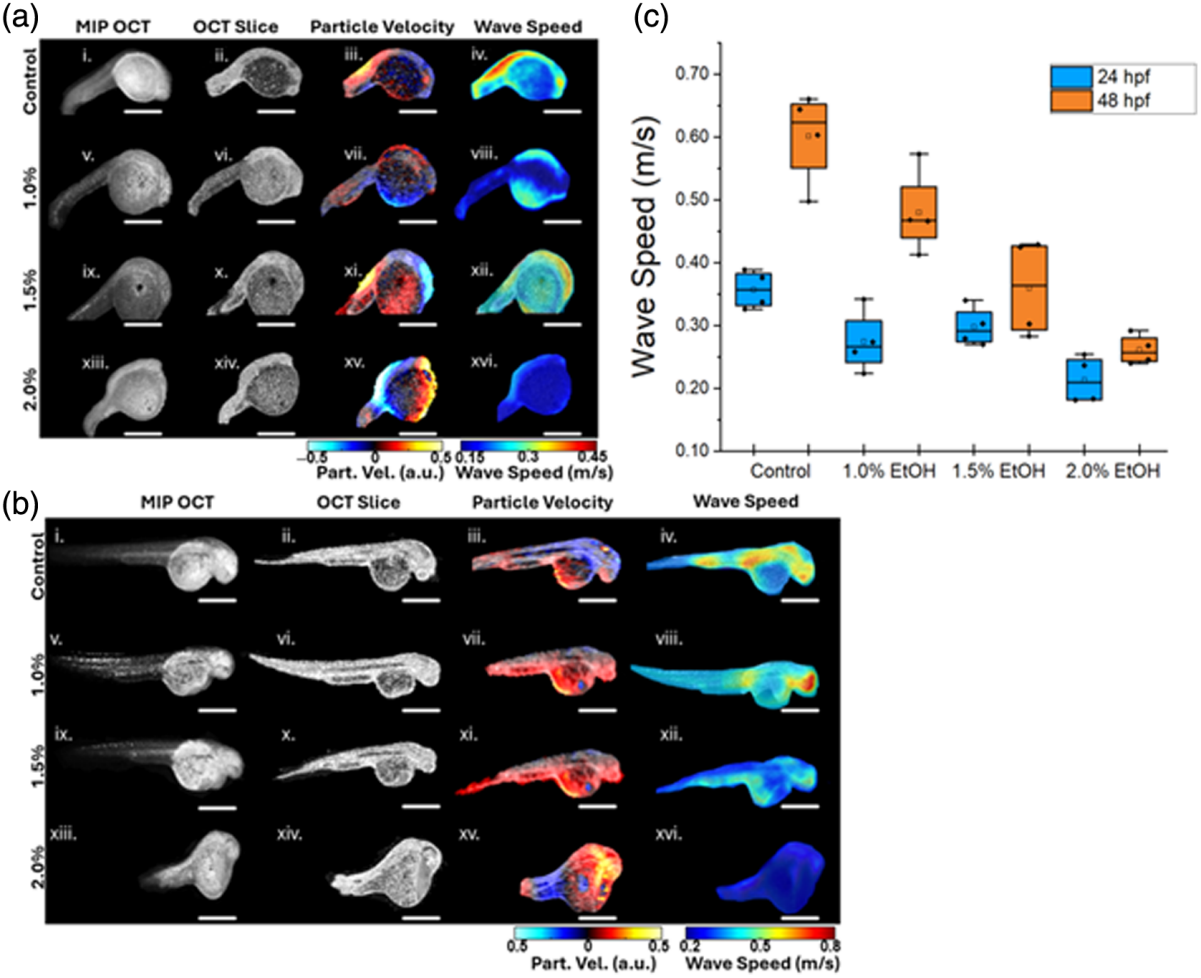
Changes in stiffness of zebrafish after ethanol exposure. OCT and OCE images at (a) 24 hpf and (b) 48 hpf for (i-iv) control, (v-viii) 1.0% EtOH, (ix-xii) 1.5% EtOH, and (xiii-xvi) 2.0% EtOH exposure groups show an MIP of the 3D OCT image, a central sagittal slice, the axial particle velocity map at 2.5 ms after excitation initiation, and the shear wave speed map. The results displayed in the figure were obtained from parallel experiments. All scale bars are equal to 500  μm. (c) Corresponding shear wave speeds obtained from OCE on the zebrafish embryos at 24 and 48 hpf, demonstrating softening of embryos after exposure to ethanol. For control and all ethanol concentrations, n=4 at 24 and 48 hpf. Statistical significance is indicated in Table 1. Data are presented as box plots, where the box is the interquartile range, the whiskers are the 25th and 75th percentiles, the central line is the median, the inscribed box is the mean, and outliers are shown as individual points.

Using OCE, we examined the impact of ethanol exposure on the stiffness of brain tissues among the zebrafish embryos at 24 and 48 hpf. Elasticity is a measure of a tissue’s resistance to deformation when an external force is applied. Stiffer materials have greater values of elastic modulus, e.g., Young’s modulus, and elastic modulus is commonly determined by measuring shear wave speed.^65^^,^^75^ A reduction in wave speed represents a decrease in stiffness, or softening.^65^^,^^75^ Typical OCT maximum intensity projections (MIPs), central sagittal slices, snapshots of the axial particle velocity of the reverberant shear wave field^80^^,^^81^ at 2.5 ms, and local shear wave speed maps are shown for each treatment group of zebrafish at 24 hpf in Fig. 6(a) and at 48 hpf in Fig. 6(b). As also shown in Fig. 6, particle velocity frames show the reverberant wave propagation within the samples for 24 and 48 hpf at a single instant. Higher shear wave speed indicates regions of increased stiffness. Statistical analysis of the changes in stiffness resulting from ethanol exposure was quantified based on the shear wave speed measurements in the brain, which were determined from the OCT structural images. Analysis by one-way ANOVA revealed significant softening of embryos’ brains at 24 hpf as a function of ethanol exposure (F=9.828, df=3, p=0.001) as shown in Fig. 6(c). Post-hoc pairwise analysis with Bonferroni testing revealed that in comparison to the control group [Fig. 6(a)(i – iv)), the difference in overall stiffness for the 1.0% EtOH [Fig. 6(a)(v-viii)] (t=3.093, p=0.559) and 1.5% EtOH [Fig. 6(a)(ix – xii)) (t=2.211, p=0.283) groups was not significant, whereas the difference in stiffness for the 2.0% EtOH group [Fig. 6(a.xiii – xvi)] (t=5.358, p=0.001) was significant. When compared with the 1.0% EtOH group, the differences in stiffness between the 1.5% EtOH group (t=0.881, p=1) and 2.0% EtOH group (t=2.265, p=0.257) were not significant. Last, when comparing the 1.5% EtOH and 2.0% EtOH groups (t=3.147, p=0.051), no significant differences were observed.

Analysis by a one-way ANOVA also indicated significant softening of embryos at 48 hpf as a function of alcohol exposure (F=21.111, df=3, p<0.0001) as shown in Fig. 6(c). Post-hoc pairwise analysis with Bonferroni testing revealed that in comparison to the control group [Fig. 6(b)(i – iv)], the difference in stiffness within the brain for the 1.0% EtOH group [Fig. 6(b)(v – viii)] (t=2.672, p=0.122) was not significant, whereas the differences in stiffness for the 1.5% EtOH [Fig. 6(b)(ix – xii)] (t=5.323, p=0.001) and 2.0% EtOH [Fig. 6(b)(xiii – xvi)] groups (t=7.495, p<0.0001) were significant. In comparison to the 1.0% EtOH group, the difference in stiffness among the 1.5% EtOH group (t=2.651, p=0.127) was not significant, whereas the difference in the 2.0% EtOH group (t=4.823, p=0.0025) was significant. Last, when comparing the 1.5% EtOH and 2.0% EtOH groups (t=2.172, p=0.304), no significant difference in brain stiffness was observed.

## 4 Discussion

### 4.1 LSFM Shows Reduced Wnt Expression

LSFM images showed that there was a disruption in development from a lack of expression of GFP throughout the CNS at 24 hpf [Figs. 4(a) and 4(c)], which was a phenotype of disrupted *wnt1* and *wnt10b* gene expression. As *wnt1* and *wnt10b* gene expressions are crucial to the activation of  Wnt/β-catenin signaling, the lack of GFP expression in LSFM images also revealed likely alterations to Wnt/β-catenin signaling. Wnt/β-catenin signaling regulates the expression of genes that are involved in the differentiation and proliferation of cells, in addition to cell survival, inflammatory response, and apoptosis.^93^ β-catenin, one of the key components in this signaling pathway, is a protein that plays a paramount role in physiological homeostasis and regulates transcription for Wnt signaling.^94^ Alcohol has been shown to alter Wnt/β-catenin signaling by downregulating Wnt/β-catenin signaling components, ultimately suppressing the pathway.^93^ As the Wnt/β-catenin signaling pathway involves feedback loops, the consequence of this downregulation is reduced mRNA expression of Wnt genes, including *wnt1*, *wnt3*, and *wnt10b*. Studies performed in both murine models and zebrafish have demonstrated that loss of Wnt genes (*wnt3*, *wnt3a*, *wnt1*, and *wnt10b*) results in a phenotype characterized by failure to develop the entire midbrain-hindbrain region.^93^^,^^95^ Furthermore, inhibition of Wnt/β-catenin signaling in zebrafish has been shown to lead to truncation of the forebrain and posterior cord syndrome (PCS), which is characterized by incomplete development of the dorsal spinal cord.^20^^,^^96^ Evidence of zebrafish embryos suffering from PCS was apparent in LSFM images, as exposure to 1.5% and 2.0% EtOH resulted in the absence of fluorescence expression beyond the hindbrain [Fig. 4(a), 4(c)]. Furthermore, embryos exhibited altered expression in the MHB at 24 hpf, which is consistent with the phenotypic effects of inhibited Wnt/β-catenin signaling. Although the 24 hpf embryos exhibited a lack of distinct MHB formation after exposure to 1.5% EtOH and 2.0% EtOH, abnormal development of the MHB was still persistent at 48 hpf, along with abnormal fluorescence expression in the hindbrain ventricle [Figs. 4(b) and 4(d)], indicating persistent effects of ethanol on neural development.

**Fig. 7:**
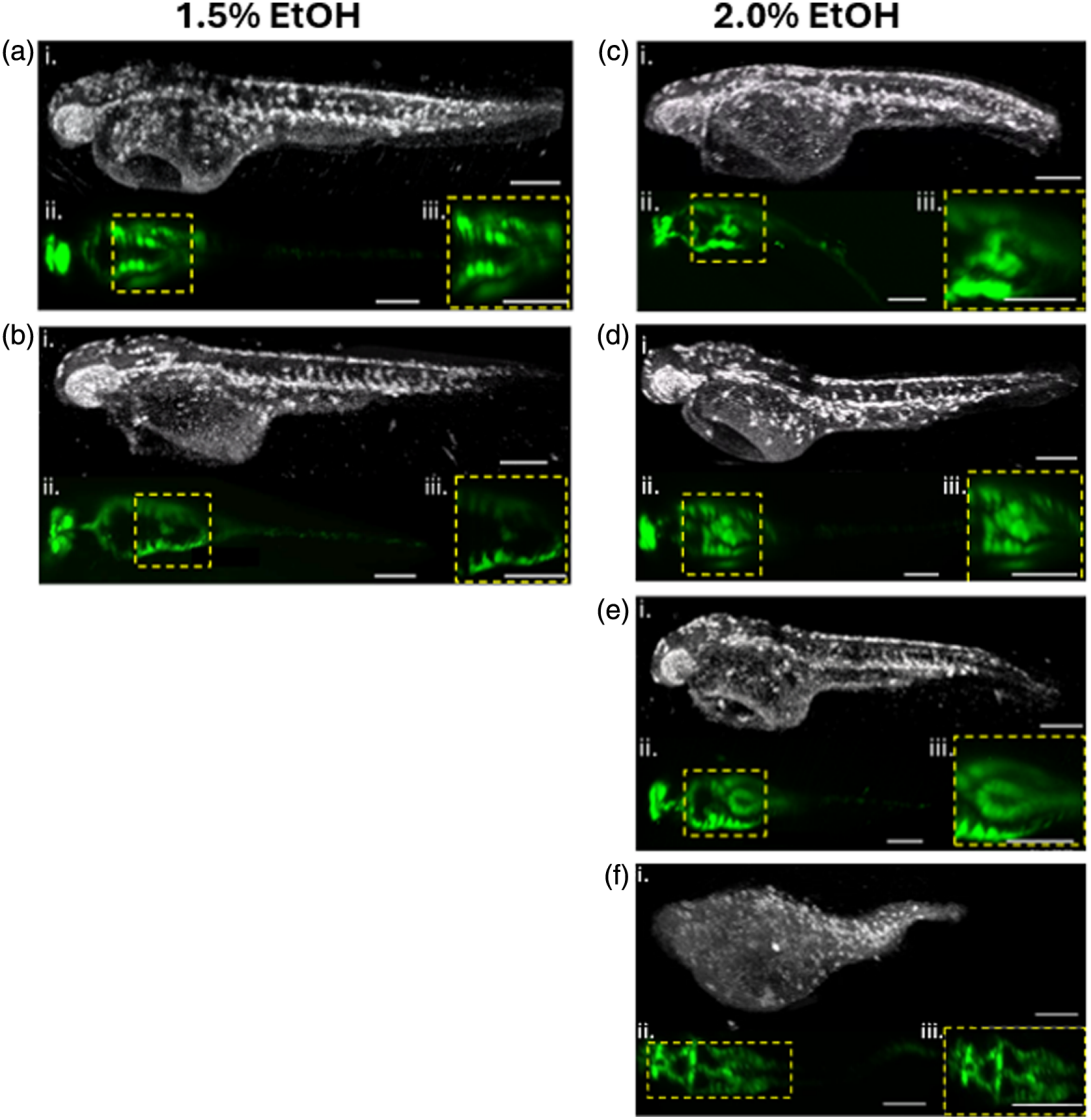
LSFM reveals hindbrain defects after exposure to ethanol, which are undetectable with OCT. (i) OCT images of zebrafish embryos at 48 hpf after exposure to (a), (b) 1.5% and (c)–(f) 2.0% EtOH. (ii) Corresponding LSFM images that show the whole embryo. Yellow dashed lines indicate the area of hindbrain ventricle defects. (iii) Zoomed in view of hindbrain ventricle defects due to ethanol exposure. The results displayed in the figure were obtained from parallel experiments. The scale bar for each image is 200  μm.

Utilizing LSFM allowed us to visualize morphological defects within the MHB and hindbrain ventricle, which were undetectable with OCT, as shown in Fig. 7. Although the embryos in the OCT images in Fig. 7 only exhibit pericardial edema and minor spinal curvature, LSFM images show abnormal MHB formation, in comparison to control embryos [Fig. 3(b)], as well as the formation of abnormal structures within the hindbrain ventricle of zebrafish embryos after exposure to 1.5% and 2.0% EtOH. The hindbrain ventricle, which is analogous to the fourth ventricle in mammalian brains, is a cerebrospinal fluid (CSF) filled cavity within the hindbrain that forms after neural tube closure.^97^ This ventricle is crucial to both brain development and function as it maintains active roles in the removal of waste, the protection of brain tissue, and the circulation of nutrients.^97^ Development and expansion of the hindbrain ventricle occurs between 17 and 26 hpf, and exposure to ethanol during this period can severely disrupt the development of the hindbrain due to neural tube closure defects.^98^ Figures 7(c) and 7(e) show embryos in which the LSFM image highlights looping within the hindbrain ventricle after exposure to 2.0% EtOH. This structural defect is the result of the failure of the hindbrain ventricle to fully inflate due to ethanol exposure, resulting in reduced ventricle size and constriction of the posterior brain. Figures 7(a), 7(b), 7(d), and 7(f) show the formation of abnormal structures within the hindbrain ventricle that are not present in the LSFM images for the control embryos. We hypothesize that the abnormalities shown are alterations of the hindbrain ventricle architecture due to F-actin and myosin II disorganization.^99^^,^^100^

Rhombomeres are segmented compartments along the neural tube in the hindbrain, which influence the development of specific cell types.^99^ This segmentation of the hindbrain ventricle is regulated by various signaling pathways, including Wnt signaling, which is active within the boundaries of rhombomeres. Expression of *wnt1* can be observed at the boundary of rhombomeres along the zebrafish hindbrain, appearing as a series of striations throughout the hindbrains of embryos in LSFM images [Figs. 3(a) and 3(b)]. Wnt has also been shown to coordinate with Tafazzin to regulate the morphogenesis of the hindbrain and hindbrain ventricle.^99^ Tafazzin, also referred to as Taz, is a protein that operates as a transcriptional co-activator to regulate gene expression, promote cell growth and proliferation, and regulate tissue generation and organ size.^101^^,^^102^ Taz mRNA is also localized at the boundaries of rhombomeres, and the stability and transcriptional activity of Taz protein in this region depend on active Wnt signaling.^99^^,^^102^

As previously stated, ethanol exposure disrupts Wnt/β-catenin signaling. This consequently alters Taz, which leads to altered morphogenesis of the hindbrain ventricle. During the initial phase of development from 17 to 24 hpf, Taz mutations phenotypically present as failure of midline separation, disturbances to F-actin and rhombomere organization, and an abnormal MHB shape.^101^ During the second phase of development from 24 to 26 hpf, Taz mutations lead to reduced ventricle size due to failure of the ventricle to fully inflate.^101^ As demonstrated in Figs. 7(c) and 7(e), embryos exposed to 1.5% and 2.0% EtOH suffered from partial failure of midline separation and failure of the hindbrain ventricle to fully inflate. Furthermore, LSFM images of embryos in the remaining subfigures show the formation of abnormal structures within the hindbrain ventricle, which are the result of disorganized F-actin and myosin II. As a result, we see that ethanol treatment contributes to hindbrain ventricle deficits possibly by inhibiting expression of Wnt genes and consequently, Taz stability. These findings parallel results from previous studies, which identified comparable hindbrain ventricle defects via *in situ* hybridizations and fluorescence imaging via stereomicroscope.^99^

Understanding ethanol-induced hindbrain developmental defects is highly important, as hindbrain ventricle malformations can result in severe neurological and physical impairment, including cognitive deficits, motor impairment due to reduced coordination and muscle tone, and potential cranial nerve dysfunctions. Furthermore, reduced ventricle size and inflation can lead to hydrocephalus due to impaired flow of CSF, causing further inflammation and damage to the surrounding brain tissue. Although OCT alone could not reveal ethanol-induced abnormalities in embryonic brain development, the integration of LSFM enabled us to capture the early onset of hindbrain malformations that would otherwise remain undetectable, potentially until later stages. This capability to detect subtle hindbrain ventricle defects showcases the critical role of LSFM in advancing our understanding of how ethanol disrupts fetal neural development and highlights the value of transgenic zebrafish as a powerful and translational model for FASD research, offering insights that would otherwise remain hidden.

Previous studies utilizing different transgenic zebrafish lines have also investigated the effect of ethanol exposure on neural development via fluorescence. Our results parallel those of previous studies as we observed that ethanol exposure alters the morphogenesis of the brain and spinal cord of embryos.^52^^,^^85^ Similar to a study that evaluated FASD in zebrafish through epifluorescence, we also observed that although some ethanol-exposed embryos do not exhibit structural abnormalities, fluorescence imaging reveals the presence of brain and spinal developmental deficits.^52^ Furthermore, through observed changes in fluorescence expression following each concentration of ethanol, LSFM also showcased that higher ethanol concentrations more severely impact Wnt gene expression, consequently altering Wnt/β-catenin signaling. Although previous studies have investigated the ethanol-induced impacts of retinal Wnt signaling, our study provides an in-depth analysis of the impact of ethanol exposure on CNS formation, which is not observable with traditional microscopy. By utilizing LSFM, we were able to perform optical sectioning and three-dimensional visualization of the embryos following ethanol exposure. Although LSFM offered valuable molecular-scale insights into zebrafish embryonic development, it is limited in its ability to visualize the morphology of nonfluorescent surrounding structures and tissues. Nonetheless, our LSFM results revealed hindbrain structural defects that would have otherwise remained undetected, emphasizing the importance of integrating molecular and structural assessments to uncover ethanol’s role in FASD. By combining LSFM with OCT, we overcame the individual limitations of each technology, enabling simultaneous, co-planar imaging of brain morphology within the context of the entire organism and visualizing brain development deficits alongside broader structural malformations. Using $\mathrm{Tg}(\mathrm{ALW}11{)}^{g7}$ zebrafish with LSFM, we identified that ethanol specifically disrupts *wnt1* and *wnt10b* gene expression, thereby altering Wnt/β-catenin signaling. This capability allowed us to directly correlate disruptions in Wnt/β-catenin signaling with changes in the biomechanical properties and morphological structures of zebrafish embryos observed through OCT and OCE, achieving a parallel molecular, structural, and mechanical assessment of ethanol-induced developmental deficits.

### 4.2 OCT Shows Structural Deformation

Our analysis of OCT images revealed that with greater ethanol exposure (1.5% and 2.0% EtOH), there was a very significant decrease in the body and notochord length among the zebrafish embryos [Figs. 5(a) and 5(b)]. We also observed that notochord development was more sensitive to ethanol exposure than body length, as exposure to a lower ethanol concentration (1.0% EtOH) resulted in a significant decrease in notochord length among the embryos. From these results, we hypothesized that the observed decrease in body length in the higher ethanol exposure groups resulted from the decrease in notochord length. As stated, alcohol disrupts Wnt/β-catenin signaling, which, during the somitogenesis period, results in posterior truncation in zebrafish embryos, supporting our findings.^20^^,^^96^^,^^103^ Comparable defects to the body of zebrafish embryos have been observed with exposure to other commonly used substances, such as nicotine^104^ and caffeine.^105^ This observation was also consistent with our results obtained through LSFM, which revealed the development of PCS among ethanol-exposed embryos, marked by the absence of fluorescence beyond the midbrain.

As demonstrated through the LSFM images, exposure to ethanol disrupts the expression of *wnt1* and *wnt10b*, consequently altering Wnt/β-catenin signaling, phenotypically displayed as reduced expression of GFP. Research has shown that Wnt/β-catenin signaling is necessary to promote pericardial proliferation and differentiation.^106^ In a study where Wnt signaling was inhibited during pericardium formation, Wnt-inhibited embryos portrayed severe pericardial edema in comparison to control embryos.^106^ When Wnt signaling is suppressed, related processes are disrupted, such as the secretion of *Sfrp1*, which is a protein involved in the regulation of cell growth and differentiation and fibroblast conversion for tissue remodeling.^107^^,^^108^ Furthermore, changes in *Sfrp1* have been linked to tissue fibrosis and alterations to both pericardial cell quantity and composition. This demonstrates that ethanol exposure can lead to pericardial edema by suppressing Wnt/β-catenin signaling, which results in the alteration of the mechanical properties of the pericardium by disrupting downstream processes such as the secretion of *Sfrp1*. This knowledge allowed us to correlate the pericardial edema observed in OCT images of embryos exposed to EtOH with the disruption to Wnt/β-signaling that was revealed through LSFM images. Supporting studies have also reported severe, irreversible pericardial edema in zebrafish embryos exposed to 2.0% ethanol,^51^ which was also consistent with our observations. Comparable effects have also been observed with exposure to other teratogens and commonly consumed substances such as nicotine^104^ and caffeine.^105^

**Fig. 8:**
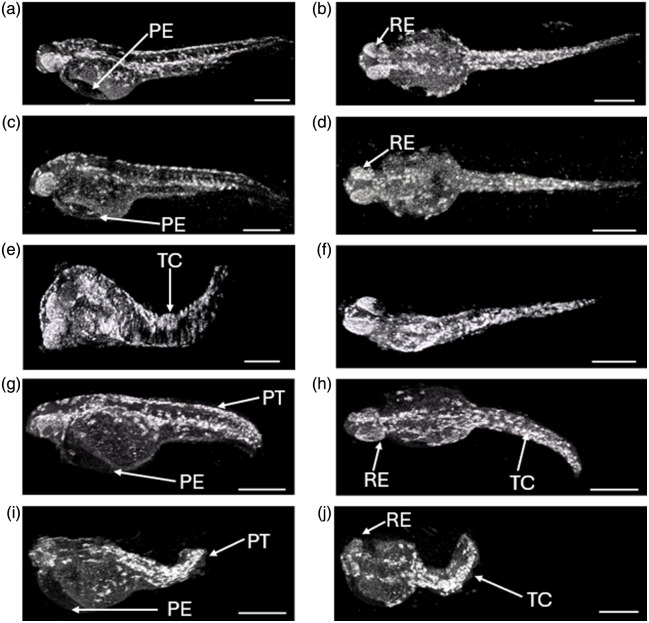
Heterogeneous phenotypes manifested in embryos exposed to the same concentration of ethanol. OCT images depict (left column) lateral and (right column) dorsal views of embryos at 48 hpf after exposure to 2.0% EtOH which exhibit different morphological defects including (a)–(d) normal body axis, pericardial edema (PE), and reduced eye size (RE), (e)–(f) malformation to the body axis and severe tail curvature (TC), (g)–(h) pericardial edema, posterior truncation (PT), and severe tail curvature, and (i)–(j) pericardial edema, posterior truncation, severe tail curvature, and reduced eye size. The results displayed in this figure were obtained from the same experiment. All scale bars are 300  μm.

Although most studies have quantified changes in eye diameter in response to ethanol exposure,^25^^,^^52^^,^^109^ we focused on changes in the average eye volume in addition to eye diameter. Like other studies, we found that exposure to ethanol resulted in a decrease in eye diameter, which as a result, led to a decrease in overall eye volume. Only exposure to 2.0% EtOH resulted in a significant decrease in eye volume among the embryos [Fig. 5(d)]. Furthermore, the changes in eye morphology were not uniform across all embryos exposed to the same concentration of ethanol (Fig. 8). For example, among the embryos exposed to 2.0% EtOH, the reduction in eye volume varied across the group (relative standard deviation = 40.74%). Although some embryos experienced a small reduction in eye volume, several embryos developed abnormally shaped eyes and even a deficiency of eyes (Fig. 8). This led to the inability to quantify the eye volumes of all embryos exposed to 2.0% EtOH [Eq. (S1) in the Supplementary Material]. Therefore, it is highly probable that the concentration of 2.0% EtOH had a more significant effect on eye volume than is represented by eye volume quantifications. Similar to pericardial development, Wnt/β-catenin signaling is crucial to eye development as it is responsible for mediating ocular tissue patterning, expression of eye-specific genes, and formation of the lens, optic cup, and retina.^96^^,^^110^ In the absence of Wnt/β-catenin signaling, expression of eye-specific genes does not occur, leading to multiple ocular malformations due to improper cell differentiation.^96^ In zebrafish embryos, disruption to Wnt/β-catenin signaling typically results in a loss of eye field markers, leading to the formation of one instead of two optic vesicles or the fusion of the eyes, i.e., cyclopia.^96^ By revealing the ethanol-induced disruption of Wnt/β-catenin signaling, molecular assessment of the zebrafish embryos via LSFM allows us to correlate the severe eye malformations observed in the OCT images with the alteration of the signaling pathway. Furthermore, all the defects observed with OCT aligned with observations reported in comparable ethanol exposure studies. However, the observations reported in previous studies were based on brightfield microscopy, which is limited to only two-dimensional information.

Our study overcomes the limitations of brightfield microscopy as OCT enabled three-dimensional imaging of the zebrafish embryos and subsequent volumetric assessment of morphological parameters. Although OCT has a limited penetration depth due to the scattering properties of biological tissues, the optically clear nature of the zebrafish embryos enabled imaging through the entire embryo without issue. As a result, OCT allowed for a more in-depth analysis of the morphological effects of ethanol on the structural development of zebrafish embryos. Although OCT is sufficient for imaging the development of the whole embryo, its resolution (typically on the scale of 10  μm) does not allow for imaging the development of specific fine structures or processes. For future work, we will combine optical coherence microscopy, i.e., OCT with ∼1  μm resolution^111^ with LSFM to overcome the resolution limitations of OCT and image the developmental changes of specific fine structures and processes, such as neural tube closure and pericardial development with subcellular resolution. Although OCT identified gross structural abnormalities in ethanol-exposed embryos, it cannot isolate their underlying causes. Integrating LSFM and OCE bridges this gap, as LSFM uncovers the molecular disruptions that drive the functional changes quantified by OCE, and the structural changes ultimately visualized by OCT.

### 4.3 OCE Shows Softening of the Brain

Alcohol disrupts many molecular and cellular processes, and consumption of alcohol alters the biomechanical properties of various tissues, including tendons,^112^ collagen bundles,^112^ bone,^113^^,^^114^ and the cornea.^115^ This alteration to biomechanical properties has been observed with both ethanol consumption and fixation. In one study, which measured the biomechanical properties of bone after ethanol fixation, the organic matrix of bone was irreversibly altered after exposure to ethanol.^116^ Other studies, which analyzed the biomechanical properties of the Achilles tendon and femurs in rats after ethanol consumption, observed tendon rupture at lower forces^117^ and increased bone fragility.^112^ Our results parallel these findings. In comparison to the control group, embryos exposed to all concentrations of ethanol exhibited softening of tissues, as indicated by the wave speed maps (Fig. 6). The corresponding wave speed maps for the 24 hpf embryos [Fig. 6(a)] highlight softening in the brain and notochord regions of the embryos. Furthermore, softening of the whole embryo was observed with exposure to 2.0% EtOH [Fig. 6(a.xvi)] in comparison to the control group [Fig. 6(a.iv)]. At 48 hpf, significant softening was observed with exposure to 1.5% EtOH [Fig. 6(b.xii)] and 2.0% EtOH [Fig. 6(b.xvi)] in comparison to the control group [Fig. 6(b.iv)], as well as in the 2.0% EtOH group when compared with 1.0% EtOH [Fig. 6(b.viii)]. Shear wave speed maps for the 48 hpf embryos [Fig. 6(c)] highlight softening of the same regions observed in the 24 hpf embryos after exposure to 1.5% EtOH and 2.0% EtOH. Furthermore, the results obtained from Rev-OCE directly correlate with the observations in both OCT and LSFM data. The embryos that exhibited softening in the Rev-OCE demonstrated morphological defects in the OCT images. Furthermore, embryos that exhibited softening also demonstrated midbrain-hindbrain defects and disrupted fluorescence expression in LSFM images.

As previously stated, alcohol exposure disrupts Wnt/β-catenin signaling, and previous studies have demonstrated that there is a direct relationship between stiffness and this signaling pathway. Expression of Wnt genes, such as *wnt1* and *wnt3a*, is dependent on the stabilization of β-catenin, which is sensitive to changes in extracellular matrix (ECM) stiffness.^113^^,^^114^ The stiffness of the ECM modulates the Wnt/β-catenin signaling pathway through the expression of these Wnt genes.^114^ This relationship between stiffness and Wnt/β-catenin signaling has also been observed in arterial stiffness^118^^,^^119^ and bone formation.^120^ Increased stiffness has also been shown to induce increased *wnt1* and *wnt3a* expression, correlating to increased Wnt/β-catenin signaling.^113^ Therefore, it is probable that the reduction in stiffness observed among the embryos exposed to ethanol led to a disruption of Wnt/β-catenin signaling, and understanding this mechanism is an avenue of our future work. Previous work, which examined the effects of ECM stiffness on β-catenin activation through *in situ* fluorescence staining, found that an increase in fluorescence intensity was observed in stiffer ECM, indicating that β-catenin activation increased in stiffer tissue.^114^ In this work, the increase in β-catenin in the stiffer ECM corresponded with increased *wnt1* and *wnt3a* expression, highlighting activation of Wnt/β-catenin signaling in stiffer tissue. The OCE and LSFM results we obtained parallel these findings. As previously stated, the expression of GFP in the $\mathrm{Tg}(\mathrm{ALW}11{)}^{g7}$ embryos is a phenotype of *wnt1* and *wnt10b* expression. Analysis of our Rev-OCE results indicates that ethanol exposure softens embryonic tissue, particularly throughout the CNS. Our molecular assessment of ethanol-exposed zebrafish via LSFM revealed the altered disruption of *wnt1* and *wnt10b* gene expression consequently alters Wnt/β-catenin signaling. This disruption was phenotypically represented by the absence of fluorescence expression throughout the brain and spinal cord of the embryos at 24 hpf, along with the development of MHB and hindbrain ventricle defects in the 48 hpf embryos. The use of Rev-OCE and LSFM in parallel allowed us to correlate the softening of the CNS measured through Rev-OCE with the developmental abnormalities observed through fluorescence imaging in the same region. As a result, pairing mechanical with molecular assessment revealed that ethanol-induced softening of the brain and spinal tissues may have contributed to altered Wnt/β-catenin signaling in the corresponding regions of the embryo. This finding demonstrates the importance of utilizing a multimodal approach for the assessment of FASD, as mechanical or molecular assessment alone could not identify the correlation between ethanol-induced effects on biological signaling and biomechanical properties.

Exposure to ethanol also results in disruptions to the biomechanical processes that control notochord development. In early developmental stages, the primary mechanical roles of the notochord are elongation and straightening of the embryo through convergent extension, in addition to elongation of the neural plate during neurulation.^60^ Elongation of the embryo requires a large exertion of force, as the notochord must push against the surrounding tissue to straighten.^121^^,^^122^ It has been observed across many species of embryos, such as frogs and chicks, that disruption to notochord development results in reduced elongation of the embryo.^60^^,^^121^ Furthermore, disruption to notochord formation correlates with a reduction in stiffness, resulting in kinking or buckling of the notochord due to the inability to support the compressive load of the surrounding tissue.^60^^,^^121^^,^^122^ Although the analysis of OCT results alone revealed bending of the notochord/tail in response to ethanol exposure, analysis via structural imaging alone is not enough to determine how ethanol influences this, along with other observed morphological defects. However, by pairing mechanical and structural assessment, we can correlate the results obtained from OCT and Rev-OCE. Here, we observed that the bending of the notochord/tail that was observed in OCT images is a possible result of the reduction in the stiffness of the notochord of zebrafish embryos following ethanol exposure, as reduced elongation and posteriorization have been reported to be consequences of reduced notochord stiffness.^60^^,^^121^ This knowledge of the impact of ethanol on the biomechanical properties of the zebrafish provided by Rev-OCE allows us to correlate the truncation of embryos observed in both OCT and LSFM with the ethanol-induced reduction of notochord stiffness, which was revealed in the corresponding shear wave speed maps. Furthermore, these observations parallel previous studies, which found a positive correlation between elongation of the notochord and elongation of the entire embryo.^60^ We also observed that regions of the embryonic brain with reduced fluorescence expression in LSFM images directly correlate with localized tissue softening detected by OCE. These results demonstrate that the ethanol-induced physical defects observed in OCT were not merely superficial structural changes but were consequences of specific molecular changes and quantifiable alterations in tissue biomechanics. These results demonstrate the vital role of Rev-OCE in this study and demonstrate that further investigation into the impact of ethanol on tissue biomechanics during development can aid in bridging the gap between molecular disruptions and physical defects. This correlation between mechanical, molecular, and structural alterations further highlights the value of multimodal assessment to better understand the genesis of ethanol-induced embryonic deficits in zebrafish.

In our study, Rev-OCE measurements were primarily focused on the brain and notochord regions of the embryo, but other studies have examined the influence of suppressed Wnt/β-catenin signaling on the stiffness of pericardial tissue. As previously stated, reduced Wnt/β-catenin signaling has been shown to induce pericardial edema.^106^ Although existing research has employed different methods for mechanical testing of pericardial edema, such as AFM, OCE has only been utilized for biomechanical characterization of heart conditions, such as myocardial infarction,^123^ as well as for the characterization of normal pericardium.^124^ OCE has many advantages in comparison to other methods of elastography and mechanical measurement. As previously stated, clinically used methods such as MRE and USE do not have the resolution needed to measure the embryos of smaller species as zebrafish or mice.^65^ Other methods, such as AFM,^71^^,^^72^ Brillouin microscopy,^66^^–^^68^ and optical tweezers,^69^^,^^70^ have finer resolutions but significantly longer imaging times, limiting the amount of the embryo that can be mapped in a timely manner, or a complete lack of 3D capabilities. In comparison, the resolution and speed of OCE are optimal for mapping the biomechanical properties of whole embryos in 3D.^77^ Furthermore, in this study, we employed Rev-OCE,^77^ which shows improved mechanical resolution and contrast in comparison to traditional wave-based OCE methods. For future studies, we plan to focus on applying the acoustic radiation force Rev-OCE^125^ for high-throughput mechanical phenotyping of zebrafish and small mammalian embryos, such as in multiwell plates, without the need for removing the samples from the wells or dechorionation.

### 4.4 Zebrafish as a Model for FASD

The zebrafish model has revolutionized biomedical research, offering unprecedented insight into vertebrate development and disease.^126^^–^^128^ With the generation of thousands of transgenic lines, zebrafish have become indispensable for investigating the development of major organ systems, including the heart, brain, vasculature, and retina. Their popularity in developmental biology stems from several key advantages, such as their rapid and *ex-utero* development, which enables direct, real-time observation of embryogenesis. This unique feature, combined with the optical transparency of their embryos, allows high-resolution imaging of developmental processes over just a few days, which cannot be accomplished with other commonly used animal models. Furthermore, the vast availability of fluorescent reporter lines makes zebrafish unparalleled for optical imaging studies, permitting molecular and structural visualization at single-cell resolution. These features have established zebrafish as a powerful model for studying the effects of teratogens such as ethanol during early development.

Zebrafish have become widely used in PAE and FASD research, enabling researchers to explore ethanol-induced developmental disruptions in a high-throughput and visually accessible manner. However, despite these advantages, zebrafish also have limitations when modeling human conditions. Unlike humans and other mammals, zebrafish embryos develop externally, which, although advantageous for imaging, does not fully recapitulate the *in-utero* environment of human gestation. In addition, in zebrafish models of PAE, ethanol is administered through the surrounding medium, making it difficult to precisely control or quantify the dose absorbed by embryos. By contrast, mammalian models such as mice offer a more physiologically relevant route of exposure via placental transfer following maternal consumption, which can be validated by blood sampling of the mother. However, there are still limitations when using murine models for PAE research. PAE research on mice can be invasive and stressful to the animal. For example, mice often limit their consumption of alcohol when permitted to voluntarily consume alcohol. Therefore, to reach the desired levels of intoxication, aggressive methods for alcohol administration are required, such as intragastric gavage, vapor inhalation, or intraperitoneal injection.^129^^–^^131^ In addition, PAE research that examines the effects of alcohol on embryos in early or middle gestational stages often requires sacrificing the embryos and the mother.^8^ These limitations of murine models further highlight the unique advantages of zebrafish over other animal models, demonstrating the vital role of zebrafish as a model for biomedical studies of embryonic development and teratogenic effects. Using the $\mathrm{Tg}(\mathrm{ALW}11{)}^{g7}$ transgenic zebrafish line, we examined how ethanol exposure disrupts Wnt/β-catenin signaling and drives fetal developmental defects. This model enabled direct visualization of ethanol-induced molecular and structural alterations and allowed us to link these disruptions to changes in tissue biomechanics. Together, these insights reinforce zebrafish as a powerful and complementary model to mammalian systems, capable of revealing developmental mechanisms underlying FASD that may otherwise remain hidden.

## 5 Conclusion

PAE is a growing global health problem and is a leading cause of preventable neurodevelopmental disorders and physiological birth defects, which are characterized as fetal alcohol spectrum disorders.^1^^,^^3^^,^^4^ PAE results in craniofacial defects and alterations to brain structure, but the exact mechanism by which ethanol causes these defects is still unclear.^132^ In this study, we presented innovative multimodal optical imaging for investigating the effects of PAE in $\mathrm{Tg}(\mathrm{ALW}11{)}^{g7}$ zebrafish, a transgenic zebrafish line in which expression of GFP is a phenotype of *wnt1* and *wnt10b* gene expression, enabling visualization of Wnt/β-catenin signaling throughout the CNS. Utilizing a multimodal optical imaging system comprised of OCT and LSFM enabled 3D, noninvasive, depth-resolved, simultaneous, and co-planar, structural, and molecular imaging of zebrafish embryos after exposure to various concentrations of ethanol. In addition, Rev-OCE allowed us to map the changes in biomechanical properties of the embryos. Integrating mechanical assessment with structural and molecular imaging allowed us to uncover ethanol-induced developmental changes that were undetected and unmeasurable via optical imaging, providing a more in-depth analysis of the role ethanol plays in inducing growth deficits in zebrafish embryos. Furthermore, integrating this imaging pipeline, which employed LSFM, OCT, and OCE, allowed us to observe changes in gene expression, the mechanical consequences of changes in expression, and ultimately, the structural consequences.

By utilizing the $\mathrm{Tg}(\mathrm{ALW}11{)}^{g7}$ zebrafish line, we were able to examine the dose-dependent effects of ethanol on regional neurodevelopment. Our findings demonstrated that ethanol leads to the disruption of *wnt1* and *wnt10b* expression and, consequently, dysregulation of Wnt/β-catenin signaling. This disruption was phenotypically characterized in LSFM images by a decrease in fluorescence expression throughout the CNS of zebrafish embryos following ethanol exposure. LSFM images also demonstrated truncation of the zebrafish brain, lack of distinct MHB formation, and several hindbrain ventricle deficits, such as failure to fully inflate and structural disorganization. Furthermore, this disruption of Wnt/β-catenin signaling resulted in a domino effect of morphological alterations to the embryos, which were observed in the complementary assessment modalities. Rev-OCE wave speed maps revealed significant softening of the brain and notochord, which contributed to the morphological defects observed in OCT images, such as the truncation of embryos, decreased notochord elongation, and severe bending of the tail/notochord. Quantifications of zebrafish embryos through reconstruction of OCT images revealed the significance of the observed structural defects, as well as the similarities to developmental defects observed in humans affected by PAE, further highlighting the integrity of zebrafish as complementary models for FASD. These results provide new biological insight through the demonstration and visualization of multiple ethanol-induced outcomes. Our results showcase how the disruption of gene expression observed in LSFM is linked to altered tissue mechanics measured by OCE and structural abnormalities imaged with OCT. This holistic view provides a deeper understanding of the mechanobiology of ethanol-induced neurotoxicity and is the initiation of further studies focused on the mechanistic understanding of these developmental defects, alterations, and disruptions.

Although the mechanisms driving the effects of PAE are highly complex, our study, which integrated structural, molecular, and biomechanical analysis, offers critical and multifaceted insight into ethanol-induced disruptions in embryonic development. Our findings highlight that no single imaging modality alone could fully capture the origins of ethanol-induced developmental defects. However, the parallel use of LSFM, OCT, and Rev-OCE allowed for a comprehensive, correlative assessment in which we identified ethanol-induced softening of embryonic tissues and associated disruption of Wnt ligand expression. This suggests that Wnt/β-catenin signaling may be a key contributor to the observed structural and molecular abnormalities. These results not only deepen our understanding of the genesis of FASD but also underscore the utility of transgenic zebrafish models and multimodal imaging in advancing FASD diagnosis and research.

## Supplementary Material

10.1117/1.JBO.31.6.066004.s01

## Data Availability

All data analyzed during this study are included in this published article (and its supplementary information files). Additional datasets generated during the current study are available from the corresponding author upon reasonable request.
