# Supplementary material for: Multimodal optical imaging for the assessment of the teratogenic effects of ethanol on zebrafish development
Source: J Biomed Opt. 2026 Jun 26;31(6):066004. doi: 10.1117/1.JBO.31.6.066004 (PMC13322611; doi:10.1117/1.JBO.31.6.066004)
Supplement: Supplementary file 1 [file JBO_031_066004_SD001.pdf]

Supplementary Information

Supplementary Methods

Morphological Assessment

For morphological assessment of zebrafish embryos in response to the various concentrations of ethanol, 3D reconstructions of OCT and LSFM data were created and analyzed in Imaris (Oxford Instruments, Abingdon, UK). Manual measurements were performed for each sample to measure changes in body length, notochord length, tail curvature, and eye volume (Fig. S1). For body length and notochord length, the length of the embryo body and notochord were traced from head to tail to measure the overall length (Fig. S1a). For eye volume, the diameter of the minor (along the optical axis) and major (orthogonal to the optical axis) axes of each eye were measured in the same manner (Fig. S1a).(94) The volume of each eye, V, was calculated by

$$V = \frac{4}{3}\pi a^2 c, \tag{1}$$

where a was the radius of the major axis and c was the radius of the minor axis. The sample-wise eye volume was recorded as the average volume of both eyes.

To quantify the degree of curvature, the angles for a straight angle (sum of angles on a straight line) (180°), an obtuse angle (180º ≥ x ≥ 90º), and a right angle (90°) were used as a reference (Fig. S1b). Therefore, greater tail curvature corresponded to angles between 90º and 120º, while less severe curvature corresponded to angles closer to 180º.

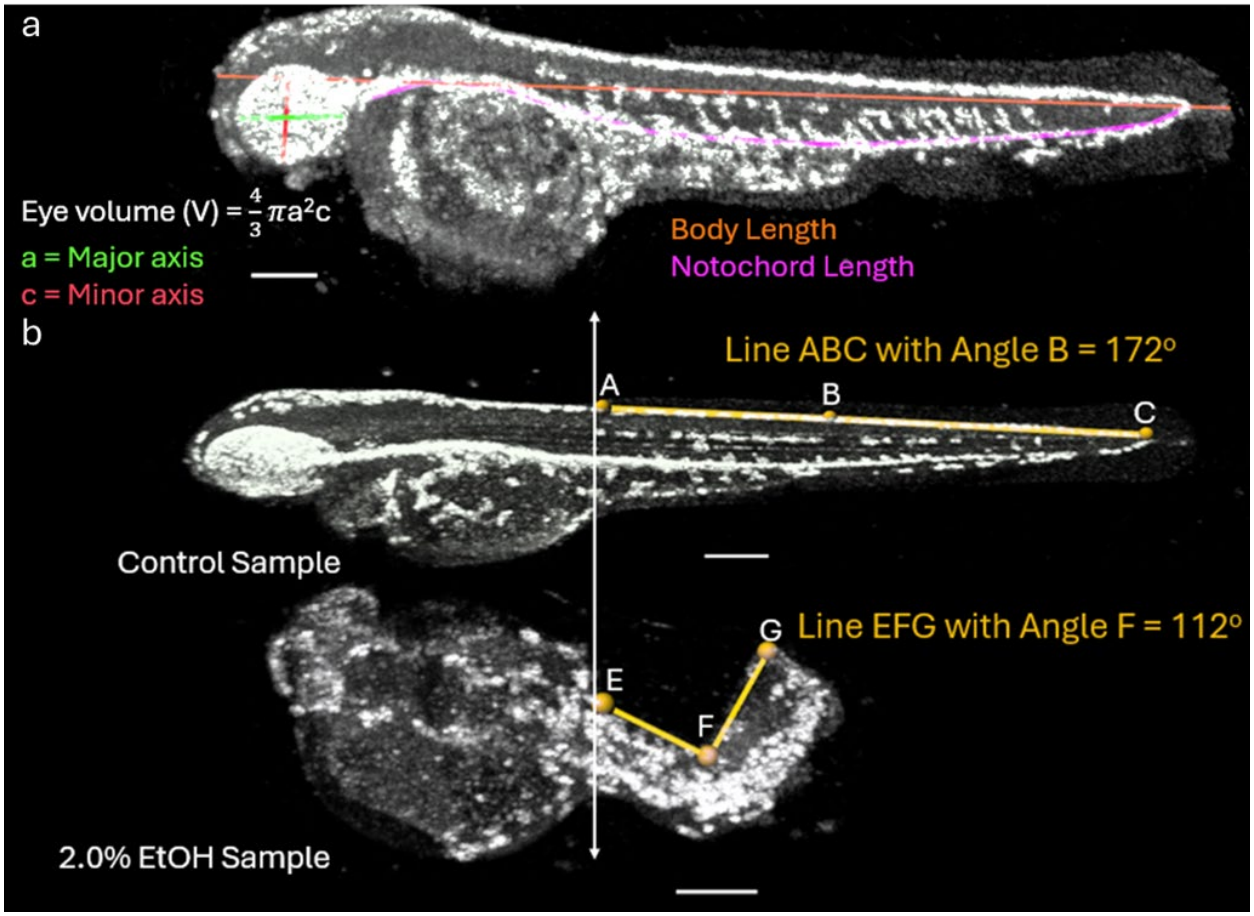

Figure S1. Reconstructions of OCT data were used to quantify morphological defects resulting from ethanol exposure. For each embryo, (a) the eye volume was calculated from the major and minor diameters of both eyes. (a) The body length and notochord length were measured from tip to tail. (b) The degree of tail curvature was calculated by drawing three points, starting from the yolk sac to the end of the tail. The angle was determined from the central point, and the amount of curvature was determined by using the angles associated with a straight angle (180°) and a right angle (90°) as a reference. Scale bars equal 200 µm.

Supplementary Results

Fluorescence Validation

Before conducting alcohol exposure studies, the fluorescence expression of the Tg(ALW11)^{g7} zebrafish embryos was validated using our LSFM system. Figure S2 shows LSFM images of Tg(ALW11)^{g7} zebrafish at 24 hpf and 48 hpf in comparison to the *in-situ* hybridization (ISH) on the Tg(ALW11)^{g7} zebrafish at the corresponding developmental stages. As expected, the observed GFP fluorescence expression occurred in the regions labeled in the ISH, indicative of Wnt ligand expression.

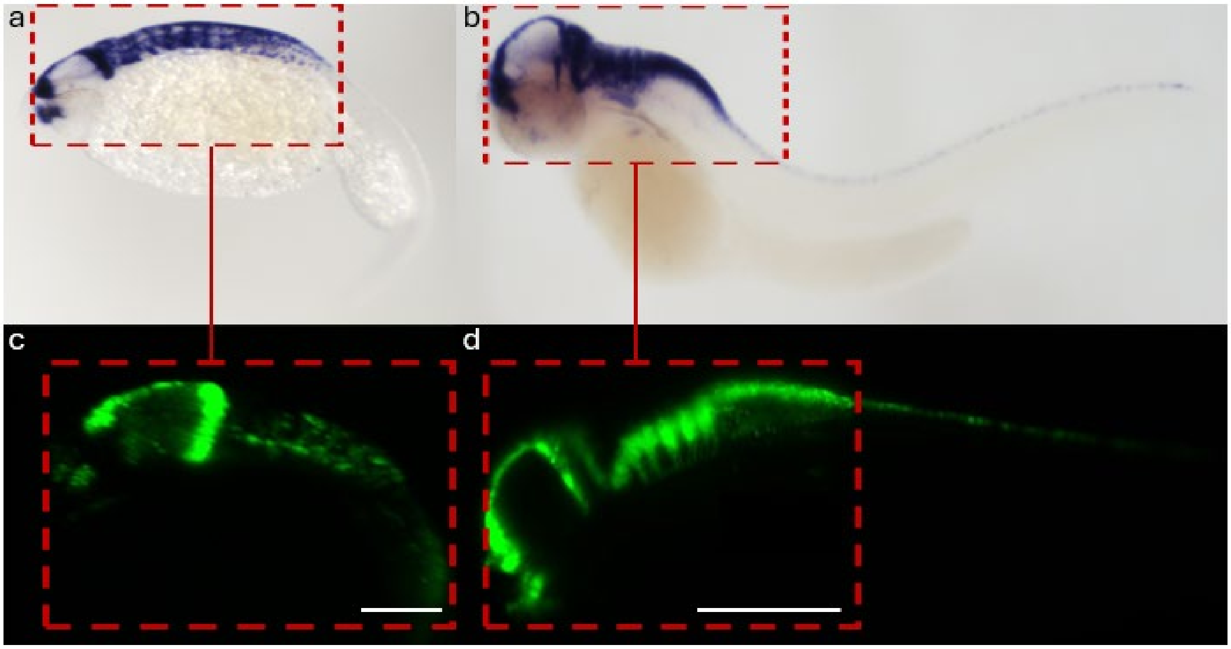

Figure S2. GFP *in-situ* hybridization on Tg(ALW11)^{g7} zebrafish. *In-situ* hybridization on the zebrafish embryos at (a) 24 hpf and (b) 48 hpf shows same pattern as fluorescence expression detected by LSFM at (c) 24 hpf and (d) 48 hpf. Scale bars equal 200 μm.

Supplementary Discussion

Mortality Rate

Previous studies have demonstrated a higher mortality rate among zebrafish embryos exposed to ethanol in a dose-dependent manner.(24) In our studies, a minimal decrease in survival was seen with an increase in ethanol concentration, however these changes were not substantial. In addition, while coagulation was observed among all alcohol exposure groups, the 2.0% EtOH exposure group possessed a greater number of embryos that suffered from no tail or a very short tail, which are all characterized as core endpoints of acute lethality.(131) Furthermore, we observed that embryos that survived until 24 hpf also tended to survive until 48 hpf, *i.e.*, survivor bias. This led to the conclusion that death due to ethanol exposure was more likely to occur within the first 24 hpf after exposure, and embryos that survived until this developmental stage had a greater chance of developing further. However, despite similar survival rates across exposure groups, the 1.5% and 2.0% EtOH exposure groups exhibited distinct, visible deformities in their body structures, such as a shortened trunk, increased pericardial size, and severe tail curvature.
